# Utilization of PMTCT services and associated factors among pregnant women attending antenatal clinics in Addis Ababa, Ethiopia

**DOI:** 10.1186/1471-2393-14-328

**Published:** 2014-09-19

**Authors:** Wakgari Deressa, Assefa Seme, Anteneh Asefa, Getachew Teshome, Fikre Enqusellassie

**Affiliations:** School of Public Health, College of Health Sciences, Addis Ababa University, Addis Ababa, Ethiopia; College of Medicine and Health Sciences, Hawassa University, Hawassa, Ethiopia; Addis Ababa City Administration Health Bureau, Addis Ababa, Ethiopia

**Keywords:** Human immunodeficiency virus, Prevention of mother to child transmission, Human immunodeficiency virus counselling and testing, Antenatal care, Addis Ababa, Ethiopia

## Abstract

**Background:**

Mother-to-child transmission (MTCT) of human immunodeficiency virus (HIV) remains the major source of HIV infection in young children. Targeting pregnant women attending antenatal clinics provide a unique opportunity for implementing prevention of mother-to-child transmission (PMTCT) programmes against HIV infection of newborn babies. This study aimed to investigate factors associated with the acceptability and utilization of PMTCT of HIV.

**Methods:**

An institution based cross-sectional study was conducted in April 2010 using exit interviews with 843 pregnant women attending antenatal care (ANC) clinics of 10 health centers and two hospitals in Addis Ababa, Ethiopia. Trained nurses administered structured questionnaires to collect data on socio-demographic characteristics, knowledge about MTCT, practice of HIV testing and satisfaction with the antenatal care services. Six focus group discussions among pregnant women and 22 in-depth interviews with service providers complemented the quantitative data.

**Results:**

About 94% of the pregnant women visited the health facility for ANC check-up. Only 18% and 9% of respondents attended the facility for HIV counselling and testing (HCT) and receiving antiretroviral prophylaxis, respectively. About 90% knew that a mother with HIV can pass the virus to her child, and MTCT through breast milk was commonly cited by most women (72.4%) than transmission during pregnancy (49.7%) or delivery (49.5%). About 94% of them reported that they were tested for HIV in the current pregnancy and 60% replied that their partners were also tested for HIV. About 80% of the respondents reported adequacy of privacy and confidentiality during counseling (90.8% at hospitals and 78.6% at health centers), but 16% wished to have a different counselor. Absence of counselors, poor counselling, lack of awareness and knowledge about HCT, lack of interest and psychological unpreparedness were the main reasons cited for not undergoing HIV testing during the current pregnancy.

**Conclusions:**

HIV testing among ANC attendees and knowledge about MTCT of HIV was quite high. Efforts should be made to improve the quality and coverage of HCT services and mitigate the barriers preventing mothers from seeking HIV testing. Further research should be conducted to evaluate the uptake of antiretroviral prophylaxis among HIV-positive pregnant women attending ANC clinics.

**Electronic supplementary material:**

The online version of this article (doi:10.1186/1471-2393-14-328) contains supplementary material, which is available to authorized users.

## Background

Mother-to-child transmission (MTCT) of HIV remains a major public health problem and continues to account for a substantial proportion of new HIV infections among young children [1]. The delivery of HIV counseling and testing (HCT) services toward pregnant women for prevention of mother-to-child transmission (PMTCT) is one of the most important HIV prevention strategies [1]. During the past decade, significant progress has been made in scaling-up PMTCT services to pregnant women, particularly in resource constrained countries [2, 3]. The risk of MTCT of HIV can be reversed through the detection of maternal infection during pregnancy and administration of antiretroviral (ARV) prophylaxis [4].

Ethiopia is one of the countries in the sub-Saharan Africa (SSA) that have a generalized HIV epidemic with about one million people living with HIV/AIDS. In 2010/11, the HIV incidence was estimated at 0.29% with an adult prevalence rate of 2.4% (1.9% among males and 2.9% among females) [5]. Women account for the larger proportion (59%) of people living with HIV/AIDS. Urban and rural HIV prevalence rates were estimated at 7.7% and 0.9%, respectively. Wide variations in HIV prevalence exist across regions, ranging from 0.9% in Somali Region to 9.2% in Addis Ababa [5].

Mother-to-child transmission (MTCT) is an important source of HIV infection among Ethiopian children. To achieve the goal of reducing the number of infants with HIV, there has been a rapid scale-up of ARV prophylaxis and HCT services in the country since 2005 [6]. All pregnant women are encouraged to access ANC services and receive information, preventive services, HCT, access to treatment and ARV for PMTCT. The national PMTCT guidelines is based on the four-pronged approaches and recommends an integrated PMTCT and HCT services within routine family planning and maternal, newborn and child, and reproductive health services at all levels [7]. Rapid HCT services are offered free of charge to all pregnant women attending routine antenatal, delivery and post-natal services in the country, using the World Health Organization (WHO) guidance on provider-initiated HIV testing and counseling (the Opt-Out) in health facilities [8].

Despite the progress made in the reduction of MTCT in Ethiopia by increasing access of pregnant women to HCT services, the proportion of pregnant women receiving the PMTCT services has been low. A total of 90,311 HIV-positive pregnant women and 14,276 HIV-positive births were reported in the country in 2010/11 [5]. However, the proportion of pregnant women counselled and tested for PMTCT was 33.4%, and only 9.3% of infants born to HIV-positive mothers received ARV prophylaxis for PMTCT.

There are a number of factors that contribute to the low uptake of PMTCT services. ANC, skilled birth attendant and HCT services influence the utilization of PMTCT for HIV. About 20% of the pregnant women in Ethiopia do not attend ANC services [5]. Even among women who attend ANC, most deliver at home due to lack of easy access to maternity services. In 2010/11, only 16.6% deliveries were attended by skilled attendant at a health institution [5]. A huge gap exists between ANC attendance and skilled attendance at birth.

Studies identified various barriers to the implementation of PMTCT of HIV including socio-economic and cultural factors affecting the uptake of HCT services, initiation of ARV prophylaxis and lost to follow up after starting ARV for PMTCT [2, 9–11]. A study conducted in northern Ethiopia found that mothers delivering at a health facility and births attended by skilled attendants were more likely to receive PMTCT services compared to those delivering at home [12]. Another study conducted in southwest Ethiopia revealed that 55% of the 426 HIV exposed infant-mother pairs did not receive any ARV prophylaxis by the mother during ANC, and mothers without ANC follow-up were five times more likely to have an infant with HIV sero positivity than those who had ANC visits [13]. The studies suggest that even HIV-positive pregnant women are identified through the ANC, many are being lost from follow up.

However, despite concerted efforts to scale-up PMTCT services in Ethiopia, the coverage and uptake of the service by the pregnant women remains low and unevenly distributed. There is surprisingly little information on the challenges and obstacles to PMTCT interventions in Addis Ababa as a result of limited studies, especially in the context of scaling up this programme [14–16]. Therefore, the aim of this study was to assess the utilization of PMTCT services and identify the possible factors associated with the service among pregnant women attending ANC clinics of public health facilities in Addis Ababa. Such information provides evidence for the identification of those factors contributing to the poor implementation of PMTCT services and fills the policy gaps towards improving HIV prevention and control services.

## Methods

### Study setting and population

This facility-based cross-sectional study was conducted among pregnant women attending ANC clinics of 10 health centers and two hospitals in the capital city of Ethiopia, Addis Ababa. The total population of the City in 2010/11 as projected based on the 2007 census was 2,975,608 [17]. Women of reproductive age (15–49 years) constitute about 32% of the total population while 2.4% of the population is estimated to be pregnant women [5]. Administratively, Addis Ababa is divided into 10 sub-cities. In 2008/09, there were a total of 11 public and 30 private hospitals, 24 public and seven private health centers and more than 550 private clinics providing health services in Addis Ababa City Administration. In each sub-city, there are public and private health facilities providing HCT for pregnant women and offering ARV prophylaxis and other necessary care for HIV positive women and their infants.

In Addis Ababa, only 68% of deliveries were attended by skilled health personnel in 2010/11 [5]. A total of 32 public health facilities in the City were providing PMTCT services at the time of the study. In 2010/11, the HIV prevalence among adults aged 15–49 years was 9.2%, with the highest prevalence of HIV infection among women (11%) than men (7.3%). The HIV prevalence rate among pregnant women attending ANC clinics was 5.8% in 2010/11 [5]. Of 3,643 HIV-positive pregnant women identified in the same year, only 46% were provided with ARV prophylaxis for PMTCT of HIV.

### Sample size and sampling procedures

The study used both quantitative and qualitative data collection methods among randomly selected 10 health centers and two purposively selected hospitals rendering PMTCT services using provider-initiated HIV testing and counseling approaches. The study population included pregnant women who received the services of ANC clinics of the selected health institutions. The sample size calculation was based on a single population proportion formula. Assuming a proportion of pregnant women attending ANC clinics of public health facilities who tested HIV-positive and received ARVs to be 50% [5], 5% significance level and 5% margin of error, the minimum sample size required for the study was 845 pregnant women after including a design effect of two and 10% non-response rate.

One health center from each sub-city was randomly selected and two hospitals, Gandhi and Zewditu Memorial Hospitals, were purposively included in the study. Gandhi Memorial Hospital mainly provides maternal health services, while Zewditu Memorial Hospital was a model hospital where HCT services were initially piloted and implemented in Addis Ababa. Sample size allocation to the health facility was proportional to the number of clients, considering average number of ANC attendants at each facility over three-months prior to the study. The selection of pregnant women for the interview was based on consecutive sampling until the sample size required for each facility was obtained.

### Data collection

All pregnant women attending the ANC clinic were invited to participate. A pre-tested structured questionnaire initially developed in English and then back translated into local language (*Amharic*) was used for data collection. The questionnaire mainly consisted of close- with some open-ended questions addressing socio-demographic characteristics, reproductive history, knowledge about the MTCT of HIV, ANC visit, waiting time at the health facility, HCT, partner’s HIV testing status, privacy and confidentiality and satisfaction with the services. Interviewers and supervisors received two-day training on the questionnaire, data collection procedures and sampling methods. Twelve trained nurses, each with assistant nurse, from each health institution administered a questionnaire using face-to-face exit interviews in April 2010. The data collection was supervised by trained supervisors with experience on PMTCT services from the School of Public Health at Addis Ababa University. A woman was eligible if she was attending ANC clinic and consented for interview. Pregnant women who were seriously ill or in labor at the time of the study were excluded.

To complement the quantitative findings, six focus group discussions (FGDs) with 6–8 participants each were held with pregnant women purposively selected from those attending the ANC clinic of the health facilities. In addition, 22 in-depth interviews with PMTCT service providers were conducted. Providers were purposively selected among those working with ANC and HIV-positive clients in the clinic, and included nurses, midwifes, clinicians, and social workers/counselors. The FGDs and in-depth interviews were conducted by trained interviewers/focus group moderators and generally lasted 1 to 1½ hours. Semi-structured FGD and in-depth interview guides were prepared in English and used in *Amharic* for data collection. All in-depth interviews and discussions were audio-recorded after obtaining permission from the participants. Both quantitative and qualitative questionnaires were adapted from the Joint United Nations Programme on HIV/AIDS (UNAIDS) best practice collection after considerable modifications were made to assess potential service barriers.

### Data analysis

Data were analyzed and reported according to the STROBE recommendations [18] (Additional file 1). All completed questionnaires and forms were checked by supervisors for completeness and consistency at field level. Data entry and cleaning was done using Epi Info version 6.04d (Centers for Disease Control and Prevention, Atlanta, GA) and analyzed using SPSS version 15 (SPSS, Inc., Chicago, IL) statistical software packages, respectively. Proportions, means, medians, frequency tables or cross-tabulations of important variables were used for data summarization and presentation. Chi square (*Χ*^*2*^) test was performed to test for statistical significance between proportions for the cross tabulated variables. Level of significance was set at p < 0.05. All tape-recorded FGDs and in-depth interviews were transcribed verbatim and translated into English by experienced translators. The written translations were checked against the tapes, and transcripts were thematically analyzed and interpreted. Qualitative data were thematically coded based on the major thematic areas of the study that include knowledge about MTCT and HCT, ANC service utilization, barriers hindering uptake of PMTCT for HIV, discussions with partners/husbands, partners HIV testing status, satisfaction with ANC/PMTCT services and healthcare providers, waiting time and cost of services. Finally, some quotes were also used in the report to complement the findings of the quantitative study.

### Ethical approval

Ethical approval and clearance for the study was obtained from the Institutional Review Board of the College of Health Sciences at Addis Ababa University. Permission to conduct the study was also obtained from the health facilities. Individual verbal informed consent was obtained from every study participant who agreed to participate in the study. All interviewers were instructed on how to comply with strict confidentiality practices for all clients both during and after data collection. All questionnaires and audiotapes were safeguarded in a secure area at the School of Public Health of Addis Ababa University for confidentiality.

## Results

### Socio-demographic characteristics of respondents

The final sample for quantitative data analysis included 843 pregnant women (99.5% response rate), and no women refused to participate in the study. About 87% (n = 734) of the respondents were from 10 health centers and the remaining 13% (n = 109) were from the two hospitals. Their age ranged from 17 to 40 years with a mean (±standard deviation (SD)) and median ages of 25.4 (±4.5) years and 25 years, respectively. About 73% of the respondents were in the age range of 20–29 years. A majority of the study participants were married (90.6%), belonged to Orthodox Christianity (68%) and Muslims (22.9%), had formal education (79%) and were housewives (60%). Among women visited the health centers, 34% had secondary or higher education compared to 62% of hospital attendants with similar education (p < 0.001). A total of 40 pregnant women and 22 service providers participated in FGDs or in-depth interviews, respectively. Most of the FGD participants had at least secondary level education with some form of college level training.

### Gestational age and reasons for visiting the ANC clinic

The average gestational age of the current pregnancy was 29 weeks (28 weeks in the health center vs. 35 weeks in the hospital), while 30% of the respondents were in their 36^th^ week or more of gestational age (Table 1). About 49% of the pregnant women were primigravida and more than half ever had two or more pregnancies. The pregnant women were asked why they visited the ANC clinic in the current health facility, and majority (93.8%) reported for ANC check-up (Table 1). About 18% and 9% of the respondents visited the ANC to receive HCT and ARV prophylaxis, respectively. Receiving ARV prophylaxis to prevent MTCT as a reason for visit was more reported in clients of hospitals (20.2%) than those in the health centers (7.2%) (p < 0.001). About 29% of the respondents visited the ANC clinic for the first time and 25% of them made such visits for four or more times during the current pregnancy. Four or more visits were more common among hospital clients (32.1%) than those in the health centers (23.7%) (p = 0.069). The average number of visits to the current ANC facility was 2.6 times, with more number of visits in hospitals than health centers.

**Table 1:** **Gestational age and reasons for visiting the ANC clinic by the type of health facility**

	Health facility		Total, n (%)
	Health center, n (%)	Hospital, n (%)	
**Gestational age in weeks**			
<12 weeks	19 (2.6)	0 (0.0)	19 (2.3)
12-24 weeks	237 (32.3)	12 (11.0)	249 (29.5)
25 weeks or more	462 (62.9)	90 (82.6)	552 (65.5)
Mean weeks	27.7	35.0	28.6
Did not know	16 (2.2)	7 (6.4)	23 (2.7)
**Number of pregnancies ever had**			
1	363 (49.5)	46 (42.2)	409 (48.5)
2	213 (29.0)	37 (33.9)	250 (29.7)
3	95 (12.9)	16 (14.7)	111 (13.2)
4 or more	63 (8.6)	10 (9.2)	73 (8.7)
**Reason for visiting the ANC clinic**			
For ANC service	693 (94.4)	98 (89.9)	791 (93.8)
For HIV testing	137 (18.7)	16 (14.7)	153 (18.1)
To receive ARV prophylaxis	53 (7.2)	22 (20.2)	75 (8.9)
For other reasons	8 (11.0)	0 (0.0)	8 (1.0)
**Number of visits made to ANC clinic**			
One	218 (29.7)	28 (25.7)	246 (29.2)
Two	168 (22.9)	23 (21.1)	191 (22.7)
Three	174 (23.7)	23 (21.1)	197 (23.4)
Four or more visits	174 (23.7)	35 (32.1)	209 (24.8)
**N**	**734 (87.1)**	**109 (12.9)**	**843 (100.0)**

### Knowledge of respondents about MTCT of HIV

A majority of the pregnant women knew about MTCT of HIV, 90.3% (95% CI = 88.1-92.2) replied that a mother with HIV can pass the virus to her child, and this knowledge was slightly higher in hospitals (94.5%) than the health centers (89.6%) although the difference was not statistically significant (p = 0.111) (Table 2). However, 22 (2.6%) of the respondents did not think that HIV could pass from mother to child, and 60 (7.1%) did not know whether or not HIV can be transmitted from mother to child. MTCT of the virus through breast milk was commonly cited by most respondents (72.4%), followed by during pregnancy (49.7%) and delivery (49.5%). Overall, 198 (26%) respondents correctly identified all the three ways of MTCT of HIV (during pregnancy, delivery and through breastfeeding). However, 91 (12%) respondents who claimed to know MTCT of HIV were unable to mention how the virus can be transmitted from mother to her infant, which was higher among health center clients than respondents from the hospitals.

**Table 2:** **Knowledge about MTCT of HIV by the type of health facility**

	Health facility		Total, n (%)
	Health center, n (%)	Hospital, n (%)	
**HIV can be transmitted from mother to her child**			
Yes	658 (89.6)	103 (94.5)	761 (90.3)
No	21 (2.9)	1 (0.9)	22 (2.6)
Didn’t know	55 (7.5)	5 (4.6)	60 (7.1)
**N**	**734 (87.1)**	**109 (12.9)**	**843 (100.0)**
Time of MTCT of HIV*			
During breastfeeding	469 (71.3)	82 (79.6)	551 (72.4)
During pregnancy	329 (50.0)	49 (47.6)	378 (49.7)
During child birth	308 (46.8)	69 (67.0)	377 (49.5)
Other	11 (1.7)	0 (0.0)	11 (1.4)
Did not know	81 (12.3)	10 (9.7)	91 (12.0)
**N**	**658 (86.5)**	**103 (13.5)**	**761 (100.0)**

^*^Multiple responses possible.

Most FGD participants knew that HIV could be transmitted from mother to child. However, only few mentioned the major ways of MTCT of HIV: during pregnancy through placenta, delivery and breastfeeding. Even, some FGD participants did not know how the transmission occurs; signaling a lot to be done to raise the knowledge of the people about HIV transmission from mother to child.

“*HIV is transmitted from mother to child during delivery and breastfeeding. During breastfeeding, we should not forcefully pull the breast out of the mouth of a child since jaws will make the nipple bleed so that a child will feed breast milk contaminated with blood. HIV is also transmitted to a child if HIV-positive mother with a sore around her mouth kisses the child”.* (28-year-old FGD participant*)*

A 35-year-old FGD participant also responded about the MTCT of HIV through umbilicus and during breastfeeding. Some FGD participants even did not know the MTCT of HIV. *I do not know that HIV can be transmitted from mother to child. I have no awareness”.* (26-year-old pregnant woman). There were also some misconceptions among some pregnant women on MTCT of HIV; they thought that the virus can be transmitted from mother to her child due to lack of sanitation.

Most FGD participants who knew how HIV is transmitted from mother to child explained that they got this information from mass media (such as television and radio) and from health care providers. The FGD participants were also asked the preventive methods of MTCT of HIV. The respondents expressed different prevention methods such as HCT during pregnancy, avoiding breastfeeding by HIV-positive women, exclusive breastfeeding during the first six months, taking medication and abiding by the advice of health professionals.

### HCT status of pregnant women

About 94% (95% CI = 92.0-95.4) of the pregnant women had been tested for HIV during the current pregnancy (Table 3). This was universally high in both health centers and hospitals, and they were tested either in the current health facility or elsewhere. About 6% of the respondents were not tested, with higher number in health centers (6.5%) than hospitals (3.7%). Psychosocial and logistical barriers were reported to prevent pregnant women from being tested for HIV. Absence of counselors, lack of awareness and knowledge about HCT, lack of interest and psychological unpreparedness, and being tested before the current pregnancy were the reasons cited for not undergoing HIV testing during the current pregnancy. Fear of being HIV-positive as a reason for not being tested was only reported by one pregnant women attending a hospital. Among pregnant women who did not test for HIV, 29 (56%) either did not know or unwilling to disclose the reason why they were not tested. Overall, 460 (54.3%) pregnant women interviewed had advised HCT to anyone else and 626 (74.3%) said they would advise someone to take the test.

**Table 3:** **Respondent’s practice of HIV testing and main reasons for being not tested by the type of health facility**

Characteristics	Health facility		Total, n (%)
	Health center, n (%)	Hospital, n (%)	
**Respondent tested for HIV in the current pregnancy**			
Yes	686 (93.5)	105 (96.3)	791 (93.8)
No	48 (6.5)	4 (3.7)	52 (6.2)
**N**	**734 (87.1)**	**109 (12.9)**	**843 (100.0)**
**Respondents’ main reason for not being tested**			
Absence of counselor	9 (18.8)	1 (25.0)	10 (19.2)
Lack of awareness and knowledge	6 (12.5)	0 (0.0)	6 (11.3)
Lack of interest and psychological	4 (8.3)	0 (0.0)	4 (7.7)
unpreparedness	2 (4.2)	0 (0.0)	2 (3.8)
Tested before current pregnancy	0 (0.0)	1 (25.0)	1 (1.9)
Fear of being positive for HIV	18 (37.5)	2 (50.0)	20 (38.5)
Did not know	9 (18.8)	0 (0.0)	9 (17.3)
No response			
**N**	**48 (92.3)**	**4 (7.7)**	**52 (100.0)**

The FGD participants expressed the importance of HCT during pregnancy to know their HIV status and to ensure that the fetus is healthy. The following quotes were taken from the FGD notes to explain the need for HCT.

*“The ANC follow-up is primarily used to know the health of the mother and fetus through HCT. Previously, the HIV testing was based on voluntarism [that is opt-in], but now all mothers are requested to be tested [that is opt-out] and this is good. If we are tested positive, we are sure that we deliver healthy baby if we use the advice and drugs properly”.* (29-year-old pregnant woman)

### HCT status of partners/husbands

With regard to partner testing, the pregnant women were asked if they knew their partner’s HIV testing status, and about 60% knew that their partners were tested (Table 4). About 35% of the respondents’ partners were not tested for HIV and the primary reason included partner’s lack of time (29%), partner’s being living in other areas (8.8%), partner’s tested before the current pregnancy (8.4%), faithfulness of partners (5.4%), lack of awareness about the importance of HIV testing (4%) and fear of the outcome (being positive for HIV) (3.4%). Partner’s lack of time for HIV testing was commonly reported among pregnant women attending health centers than hospitals. A significant proportion of the respondents did not know why their partners were not tested (22.6%).

**Table 4:** **Distribution of respondent’s partner’s with regard to HIV testing and main reasons for being not tested by the type of health facility**

Characteristics	Health facility		Total, n (%)
	Health center, n (%)	Hospital, n (%)	
**Respondent’s partner tested for HIV during the current pregnancy**			
Yes	425 (57.9)	77 (70.6)	502 (59.5)
No	272 (37.1)	25 (22.9)	297 (35.2)
Did not know	37 (5.0)	7 (6.4)	44 (5.2)
**N**	**734 (87.1)**	**109 (12.9)**	**843 (100.0)**
**Main reason’s cited for respondent’s partner for being not tested**			
Lack of time (busy)	83 (30.5)	3 (12.0)	86 (29.0)
Out of Addis Ababa or the country	21 (7.7)	5 (20.0)	26 (8.8)
Tested before the current pregnancy	24 (8.8)	1 (4.0)	25 (8.4)
Faithfulness	17 (6.3)	0 (0.0)	17 (5.7)
Lack of awareness or knowledge	10 (3.7)	2 (8.0)	12 (4.0)
Fear of being positive for HIV	10 (3.7)	0 (0.0)	10 (3.4)
Has a plan to be tested	8 (2.9)	0 (0.0)	8 (2.7)
Not willing (or interested)	4 (1.5)	1 (4.0)	5 (1.7)
Fear of stigma and discrimination	4 (1.5)	0 (0.0)	4 (1.3)
Fear of rejection by wife	4 (1.5)	0 (0.0)	4 (1.3)
Other	1 (0.4)	1 (4.0)	2 (0.7)
Did not know	60 (22.1)	7 (28.0)	67 (22.6)
No response	26 (9.6)	5 (20.0)	31 (10.4)
**N**	**272 (91.6)**	**25 (8.4)**	**297 (100)**

About 81% (n = 682) of the pregnant women interviewed said they had discussions with their partners/husbands about issues related to ANC services and HIV testing during pregnancy. More number of literate pregnant women (84.2%) than illiterate ones (68.4%) reported discussions with their partners (p < 0.001). Of the total respondents, about 98% thought that their partners/husbands had positive attitudes towards ANC services and HIV testing during pregnancy. A very small number of respondents (2.2%) reported fear of disapproval by their partners for being tested for HIV.

### Waiting time by pregnant women for ANC/PMTCT services

Time spent on waiting and discussion with ANC/PMTCT counselor was assessed among pregnant women, and the average waiting time spent was more than half an hour. More than half of the respondents (53.8% in the health centers and 41% in the hospitals) spent between 10 to 30 minutes before seeing the counselor (Table 5). The average waiting time for health centers was 37 minutes compared to 47 minutes at hospitals. The average time spent with the counselors was about 12 minutes for the entire respondents. More than two-thirds of the respondents reported 5–10 minutes of time spent with counselors, and the average time spent for participants from health centers (11.6 minutes) was shorter than the time spent for hospital clients (13.2 minutes). Overall, the time spent for waiting was significantly higher than the time spent for consultation with service providers. About 58% of the respondents justified that the time they spent during the visit was reasonable, and 29.4% of the hospital respondents compared to 15.3% from health centers reported that the time they spent during the visit was too long (p < 0.001).

**Table 5:** **Time spent by pregnant women for waiting and with counselor by the type of health facility**

Time spent in minutes	Health facility		Total, n (%)
	Health center, n (%)	Hospital, n (%)	
**Waiting time to see the counselor**			
Less than 10 minutes	103 (14.5)	20 (18.7)	123 (15.1)
10-30 minutes	382 (53.8)	44 (41.1)	426 (52.1)
31-60 minutes	134 (18.9)	23 (21.5)	157 (19.2)
61-90 minutes	30 (4.1)	4 (3.7)	34 (4.2)
More than 90 minutes	61 (8.6)	16 (15.0)	77 (9.4)
**N**	**710 (86.9)**	**107 (13.1)**	**817 (100.0)**
**Time spent with counselor**			
Less than 5 minutes	21 (2.9)	2 (1.9)	23 (2.8)
5-10 minutes	500 (70.2)	63 (58.9)	563 (68.7)
11-30 minutes	186 (26.1)	40 (37.4)	226 (27.6)
More than 30 minutes	5 (0.7)	2 (1.9)	7 (0.9)
**N**	**730 (87.4)**	**105 (12.6)**	**819 (100.0)**
**Amount of time spent during the visit**			
Too long	112 (15.3)	32 (29.4)	144 (17.1)
Reasonable (just right)	443 (60.4)	45 (41.3)	488 (57.9)
Too short	179 (24.4)	32 (29.4)	211 (25.0)
**N**	**734 (87.1)**	**109 (12.9)**	**843 (100.0)**

### Barriers to ANC/PMTCT services

Most FGD participants discussed different barriers that prevent proper utilization of ANC/PMTCT services at the health facility. The way health workers treat the mothers (mistreatment), the time taken to get ANC service (long waiting time, long time to get laboratory tests and obtain results), and client load to the health facilities were among the barriers that contribute to the low utilization of ANC/PMTCT services.

“*They [health workers] are not punctual. We hear from outside about their hospitality, but we observe them in the health center while they are insulting mothers. They waste the time by talking in a group rather than giving a proper service”.* (24-year-old pregnant mother)

Some FGD participants were unhappy with the knowledge and skill of service providers. Lack of competent care provider at health center was identified as one of the barriers affecting the quality of ANC/PMTCT services. As described by one pregnant woman who blamed a health center for lack of a doctor and visited hospital for her antenatal visit:

“*I do not think I am satisfied with the service of the health center since they do not have specialist doctor who can properly examine and treat the pregnant women. Due to lack of a specialist doctor in the health center, I came to this hospital to get a better service”.* (25-years-old pregnant mother)

Most key informants agreed that physical inaccessibility of health service, inadequate health facilities that provide maternal health services, lack of information and limited community awareness about the ANC/PMTCT services, health care provider’s attitude and economic factors are some of the main barriers to maternal health service utilization. They emphasized the need to have continuous and sustainable awareness creation of the community.

*“Our sub-city has the largest population size. There is only one public health institution rendering delivery and PMTCT services in the sub-city. Provision of maternal health services including PMTCT to these populations by one health center is very difficult.”* (Service provider)

In some cases, the physical layout and rooms of the health facilities caused difficulties. Those attending for other services overhear or identify PMTCT clients, who are then often presumed to be HIV-positive. Inadequate facilities particularly at the health center were mentioned by one of the FGD participants:

“*There are situations in which both mothers and children are served in a single room by the same service provider. Rooms should be separated for mothers and children, and there should be separate service providers for children, mothers and other patients”.* (22-year-old pregnant mother)

Majority of the FGD discussants were happy with the cost of services provided at public health facilities (hospitals and health centers). However, they were dissatisfied with the long-waiting time to receive services, poor competency of health care providers at health centers and the way they treat the clients. The following quote deals about the impreciseness of the estimated date of delivery.

“*When you go to the health center for delivery, the health workers send you back home saying that the time of delivery is not yet near. However, the mother gives birth at home before the date of delivery estimated by the health worker. There should be well trained and qualified personnel at the health center”.* (22-year-old pregnant mother)

Most FGD participants explained that the quality of ANC services at private health facilities is better than the public services. They reported that private clinics give priority to clients for immediate intervention and do not keep clients to wait for long time. However, they all agreed that private health facilities are very expensive and their services are unaffordable for most people. Had it not been for the cost of services, most FGD participants preferred the services of private health facilities than public ones.

*“I would prefer to go to a private health facility than a public health center even if it is expensive. There is a long queuing in public health centers as the service is free of charge. When you are weak, the private care providers facilitate your registration to give you immediate service. They give you much care. However, it is your obligation to process your registration and wait for your turn at public health centers even if you are weak. In the health center, no one can support and guide you if you do not have your own person accompanying you”.* (A 22-year-old pregnant mother)

In this study most FGD participants agreed that time, quality of service (satisfaction with services), service fee and attitude of the health care provider (good hospitality) were mentioned as important factors affecting maternal health service such as ANC/PMTCT service utilization. Most of them agreed that health care service providers need to respect the dignity of individuals and treat them professionally. They emphasized that health care providers don’t have to insult or mistreat mothers. One of the FGD participants emphasized the need to assign responsible health workers who provide ethically sound health services to mothers.

*“I saw a husband and wife looking for nurses for immediate service because the fetus was not moving and the mother was very weak to the extent that she was unable to speak. However, a nurse shouted on him [husband] saying that “please go away, we are busy and wait your turn”. Finally, the nurse left them and went for lunch saying the time for service is over”.* (26-year-old pregnant mother)

In the current study, the service providers have identified some critical barriers to the implementation of PMTCT service in Addis Ababa, including the difficulty of making decision on the initiation of ARV by HIV-positive pregnant mother due to lack of partner’s involvement during HCT. The service providers acknowledged the difficulties of engaging men in HCT services and emphasized the need to involve men if uptake of PMTCT service is to be improved:

*“Mothers fear to be tested alone and there is a problem on how to include their partners/husbands in this programme. We give appointment and invitation letter to their partners to come together but the partners usually do not come for HCT. Some mothers change their address and disappear after they learned that they are HIV-positive due to the fear of stigma and discrimination”.* (Service provider)

The service providers also identified the difficulty of HIV-positive pregnant women to disclose their sero-status to their partners due to fear of divorce because of their economic dependence on men.

*“Most women who tested positive for HIV do not disclose their sero-status to their partners due to fear of divorce, stigma or violence. This is something related to female’s economic dependence on male partners. As a consequence, they [women] are unwilling to take the ARVs at all or take it secretly and incorrectly”*. (Service provider)

The service providers also mentioned about the problem of PMTCT trained staff turnover and an increased workload among staff resulting in low performance of PMTCT services. This is particularly true for those health care providers working in ANC/PMTCT and labor wards.

*“There is a high turnover of people trained on PMTCT, there will be a gap when these people leave their job until new staff are trained to replace them. Some staff members consider PMTCT as an additional service for which they are not very much responsible.”* (Service provider)

However, most service providers agreed that prospective changes have been observed with regard to the uptake of PMTCT service in the recent years. Most pregnant women and their families were not as resistant as when PMTCT was initially introduced due to continued advocacy and awareness creation activities to change the attitude of people towards HIV/AIDS prevention and control. A service provider from one of the health centers reported that most ANC attendees currently volunteered to be tested for HIV during ANC visit. This was partly achieved through continuous education of clients and also sending invitation letter to their partners/husbands to discuss about the current pregnancy and related issues.

*“To increase PMTCT service utilization, mother-to-mother group established in the community is progressing well. Mother-to-mother group includes HIV-positive or negative mothers volunteered for teaching the community about PMTCT. To trace HIV-positive mother defaulters and exposed infants, nurse health extension workers are assigned in their communities. The group also performs HCT in the community”.* (Service provider)

Overall, the service providers mentioned physical inaccessibility of health facilities, lack of separate and adequate space for PMTCT services, lack of confidentiality on HCT, inadequate maternal health services, lack of information and limited community awareness about the existing services, poor attitude of health care providers and economic factors as major barriers to the uptake of ANC/PMTCT services. The need to have continuous and sustainable awareness creation of the community about the availability and use of the services were emphasized.

## Discussion

This study highlights a number of issues useful for understanding factors associated with the uptake of PMTCT services integrated into routine ANC programmes at public health facilities in Addis Ababa. The study also demonstrated potential areas for improving PMTCT interventions as part of the ANC services. In this urban setting, pregnant women went to ANC clinics for check-ups, HIV testing and to receive ARVs to protect their new born babies from the virus. Nearly 24% of the respondents visited the ANC clinic four or more times during the current pregnancy. A study conducted in Dire Dawa found that women who attended two or more ANC follow-ups at a hospital were about three times more likely to accept HCT than those with lower follow-ups [19]. Such frequent visits provide an opportunity for teaching mothers about MTCT and identifying HIV-infected women for PMTCT interventions.

The knowledge of the study participants about MTCT was quite high (90.3%). In a study done in a similar setting, 90% of postnatal mothers who delivered in Tikur Anbessa and Zewditu Memorial hospitals knew that HIV can be transmitted from an infected mother to her child [14]. This high level of knowledge may be attributed to various health education programmes being conducted both at health-facility and community levels and a broadcast through mass media in this urban setting. The women’s knowledge about MTCT in the present study was better than the knowledge reported in other African settings. In a health-facility study in Uganda, 80% of the women knew that a mother with HIV can transmit the virus to her child [9], similar to the knowledge (79%) of the pregnant women studied at ANC clinic in Khartoum, Sudan [20] and 70% in rural districts of Zimbabwe [21]. The high level of knowledge of mothers about MTCT is very critical for preventing the transmission of the virus from HIV-positive women to her child, and programmes should utilize various means of increasing the awareness and knowledge of the community through proper IEC/BCC interventions.

Prompt identification of HIV-infected pregnant women is essential for effective implementation of PMTCT services. The study showed that counseling about PMTCT and HIV testing was done during the current pregnancy for the majority of pregnant women (94%) participated in this study. Since HCT services had been recently expanded to more public and private health facilities and uptake of counseling and testing in ANC increased in Addis Ababa, the results are expected. However, studies that reported the rate of HCT among pregnant women attending ANC clinics and the subsequent PMTCT services in Ethiopia are rare, and most studies focused on HIV counseling and its quality rather than the actual practice of HIV testing and ARV prophylaxis for HIV-positive women [14–16]. A routine antenatal HIV testing among pregnant women in urban Zimbabwe showed that almost all women were tested for HIV using the opt-out strategy [22], and uptake of HCT among hospital ANC clients was reported to be 68% in western Kenya [23].

Though PMTCT service is known to reduce the transmission of HIV from mother to child its use has been limited because of various barriers [11, 24]. This study has revealed multiple social, cultural, economic and physical barriers that might hinder the success of HCT – an entry point for the PMTCT programme in both health centers and hospitals. Poor awareness and knowledge, shortage of trained staff on PMTCT, low involvement of male partners, psychological unpreparedness to accept HIV-positive result, fear of disclosure of HIV-positive status to partner, stigma and discrimination, financial and transportation problems were the main barriers identified in preventing mothers from HCT in this study. A study in Vietnam found that lack of knowledge and information due to poor counseling, and fear of stigma and discrimination were the main barriers in accessing PMTCT services for HIV-positive women [25]. Efforts are needed to address barriers that the pregnant women may face in accessing and using PMTCT services.

The support of women by husbands in ANC has several implications for the uptake of PMTCT services. This study demonstrates that spousal disapproval of HCT for pregnant women was reported to be negligible in the quantitative findings, but it was commonly reported in the qualitative data. In other studies, husbands/partners attitude towards HIV testing are important predictors of whether the mother had to test for HIV or not. A health facility data on HCT for sub-cities in Addis Ababa showed that the percentage of partners tested for HIV decreased from 6.4% in 2004 to 5.3% in 2009 [26]. About 20% of the pregnant women in Uganda reported that their husbands disapproved mother’s decision to test for HIV [9]. Similar to the present findings, several studies indicate that women’s major concern about HCT is the reaction of their male partners towards HIV-positive test result and the subsequent low rates of HIV sero-status disclosure [10, 11, 27–29].

The decision to involve male partners in the maternity services plays an important role in the uptake of ANC/PMTCT services. The strategy to inform the male partners about PMTCT services and inviting them to ANC clinic with their female partners to increase the uptake of HCT is currently implemented in many countries [1]. Most of the pregnant women interviewed in this study reported that 60% of their partners were tested for HIV during the current pregnancy. In urban Rwanda and Zambia, although 91% and 47% of the couples, respectively, were willing to test jointly, stigma and fear of partner reaction were the main barriers preventing couples from getting tested for HIV together [29]. Studies done in Malawi found that most HIV-positive women who attended follow-up visits after delivery had partner support than women who dropped out [10, 11].

Difficulty of deciding to initiate ARV by pregnant mothers due to lack of male involvement during HCT has been considered as one of the critical barriers to uptake of PMTCT services. Majority of the women tested for HIV may decide not to disclose their HIV sero-status to their male partners due to consequences including divorce, domestic violence or for women to be abandoned by their husbands and families. Therefore, a strategy should be sought to involve male partners in HCT through raising awareness, encouraging couple counseling and testing, advocating and promoting HCT and PMTCT, and reducing stigma and discrimination.

In this study, time spent for waiting was significantly longer than the time spent for consultation with service providers. The average time for waiting and consultation was shorter at health centers compared with the time spent for waiting and consultation at hospitals. A study in Addis Ababa health centers showed that the shorter the time spent for counseling leads to brief and shallow understanding of the PMTCT messages [15]. Client satisfaction is one of the factors affecting utilization of ANC/PMTCT services. In this study, about 58% of the pregnant women interviewed reported that the amount of time spent during the visit was reasonable, and several studies found that quality of communication skills, comprehensiveness of counseling information, technical competence of the counselor and privacy during counseling are the most important factors affecting the quality of PMTCT services [15, 16, 24, 30, 31]. In order to improve acceptability and uptake of the ANC/PMTCT services, consideration should be given to improve quality of the services.

Almost all PMTCT services in Ethiopia are implemented within hospitals and health centers mainly located in larger cities and towns. ANC coverage, particularly in rural areas, is very low and home deliveries by traditional birth attendants or supported by family members or relatives are very common not only in rural Ethiopia but also in urban areas. Community-based PMTCT programmemes are very critical for the success of PMTCT services and the participation of community members such as mother-to-mother groups can help to reach more people in teaching about PMTCT programme and tracing default HIV-positive mothers and exposed infants. The current launch of urban health extension workers in Ethiopia can bring about valuable benefits to community-based PMTCT interventions and open ways for a number of HIV prevention and control activities [32]. In rural Cameroon, trained traditional birth attendants offered effective PMTCT services, including rapid HIV testing [33] and more than 75% of traditional birth attendants in Zimbabwe were willing to participate in all activities that constitute the basic package of PMTCT services [21].

This study has several limitations. First, the study was based at the health facility and the pregnant women do not represent the general populations of Addis Ababa because only mothers seeking ANC at a health unit were eligible for the study, making hard to make inferences. Second, pregnant mothers were selected based on consecutive sampling strategy which is not based on random selection. Third, nurses and midwifes working in the health facilities administered the questionnaires and this may have led to social desirability bias. Fourth, the findings may be affected by recall bias since mothers were interviewed about their knowledge and acceptance of HCT, but were not observed to determine the ultimate acceptance of HIV test. Finally, this study focused on HCT, and the HIV-positive women were not identified and followed to determine those who eventually receive ARV prophylaxis to reduce the risk of HIV transmission to their infants and antiretroviral therapy for their own health. Despite these limitations, the study identified a number of barriers that can hinder proper uptake and implementation of PMTCT services among ANC attendees. These barriers can be easily mitigated if properly planned and addressed.

## Conclusion

This study revealed that HCT among ANC attendees and knowledge about MTCT of HIV was quite high in both health centers and hospitals. Male partners’ HCT during the current pregnancy was reported to be acceptable. Understanding factors associated with HCT services is an important step toward improving the implementation of the interventions. Lack of awareness and knowledge about the availability and benefits of ANC/PMTCT services, shortage of PMTCT service providers, lack of adequate and separate room for PMTCT services, poor involvement of partners/husbands in ANC/PMTCT services, poor disclosure of HIV-status to partners and psychological unpreparedness due to fear of being positive for HIV were the main barriers preventing mothers from HIV testing. Although the majority of pregnant women reported their satisfaction with the sessions they had with counselors, efforts should be made to improve the quality of the HCT services. ANC attendance and HCT soon after recognition of pregnancy and awareness about PMTCT services should be promoted through training and education of health extension workers and community-based volunteers. In order to improve implementation of PMTCT services, further research should be conducted to evaluate the uptake and effectiveness of ARVs among HIV-positive pregnant women attending standard of care at ANC clinics.

## Electronic supplementary material

Additional file 1: **STROBE Statement—Checklist of items that should be included in reports of** ***cross-sectional studies.*** (PDF 99 KB)

## Abbreviations

AIDS: Acquired immune-deficiency syndrome
ANC: Antenatal care
ARV: Antiretroviral
FGDs: Focus group discussions
HCT: HIV counselling and testing
HIV: Human immunodeficiency virus
MTCH: Mother-to-child transmission
PMTCT: Prevention of mother-to-child transmission
SD: Standard deviation
SSA: Sub-Saharan Africa
UNAIDS: Joint United Nations programme on HIV/AIDS
WHO: World Health Organization.
